# Platelet receptors; an instrumental of platelet physiology

**DOI:** 10.12669/pjms.293.3497

**Published:** 2013

**Authors:** Muhammad Saboor, Qamar Ayub, Samina Ilyas, Moinuddin

**Affiliations:** 1 Muhammad Saboor, Baqai Institute of Hematology, Baqai Medical University, Karachi, Pakistan.; 2 Qamar Ayub, Baqai Institute of Medical Technology, Baqai Medical University, Karachi, Pakistan.; 3 SaminaIlyas, Baqai Institute of Hematology, Baqai Medical University, Karachi, Pakistan.; 4 Moinuddin, Baqai Institute of Hematology, Baqai Medical University, Karachi, Pakistan.

**Keywords:** Platelet receptors, Integrins, Selectins, Tetraspanins

## Abstract

Platelets play an important role in hemostasis, inflammation, host defense, tumor growth and metastasis. Platelets receptors are instrumental in platelet-platelet aggregation and interaction of platelets with leukocytes, endothelial cells and coagulation factors. These receptors are also the targets for antiplatelet drugs. This review focuses on the role of platelet receptors in human physiology. Data were extracted from peer-reviewed journals using MEDLINE and EMBASE databases, and the following terms (platelets, platelet receptors, CD markers, integrins, tetraspanins, transmembrane receptors, prostaglandin receptors, immunoglobulin superfamily receptors) were used.

## INTRODUCTION

Platelet receptors are important for normal functioning of the platelets. These receptors either activate platelets or they act as adhesion molecules which interact with the damaged endothelium, other platelets and leukocytes. Platelets are not only instrumental in hemostasis, they also have a role in inflammation, antimicrobial activity, angiogenesis, tumor growth and metastasis.^1^^-^^4^ Platelets are unable to perform these functions in the absence of their receptors. Disorder of platelet receptors were first described by Glanzmann in 1918 and Bernard in 1948. After the recognition of these disorders, structure and functions of platelet receptors were extensively studied. Some of the well-recognized platelet receptors are; integrins, leucine-rich repeats receptors, selectins, tetraspanins, transmembrane receptors, prostaglandin receptors, lipid receptors, immunoglobulin superfamily receptors, tyrosine kinase receptors and miscellaneous platelet receptors.

***1. Integrins*** ***: ***Integrins are adhesion and signaling molecules that are present on many types of cells including platelets. These are non-covalently bound heterodimers of α and β subunits. Platelets have three types of integrins i.e. β1, β2 and β3.

β1 family has three members i.e. α2β1, α5β1 and α6β1.

α2β1 receptor or GPIa-IIa is an important platelet receptor for collagen. Number of α2β1 receptors present on platelet surface varies from 2000-4000. α_2_ subunits contain an I domain for Mg2+ions that provides the critical site for interaction with collagen.^5^ α2β1 receptors promote platelet adhesion to collagen, stabilize thrombus growth and promote pro-coagulant activity. They also initiate synthesis of platelet proteins after binding with collagen.

α5β1 receptors play a complimentary role in platelet functions; they facilitate platelet adhesion to fibronectin.^6^

α6β1 integrin mediates platelet adhesion to laminin which is found in the basement membranes and extracellular matrix. Magnesium and manganese and cobaltact as cofactors for this interaction.^6^ α6β1 signaling to the platelets via phosphoinositide 3 kinase induces morphologic changes in the platelets.^7^

***β2 family***

αLβ2(CD102) is also known as intercellular adhesion molecule 2 (ICAM-2). It is the only known member of β2 integrin family. It has approximately 3,000 copies on the surface of the platelets and also on the open cannlicular system. It is considered important for platelet adhesion to neutrophils and also for platelet-leukocyte interaction.^8^

***β3 family***

αIIbβ3 (CD41/CD61)also known as GPIIb-IIIa complex, is present only on the platelets and is the most abundant platelet adhesion receptor. Resting platelets express 80,000-100,000 molecules of αIIbβ3 on their surface.^9^ In addition to the surface receptors, 20,000-40,000 molecules of αIIbβ3 are also present in the α-granules, dense granules and on the membranes of open canalicular system of the platelets. These receptors become translocated to the platelet plasma membrane after activation and release reaction.

Upon platelet activation, αIIbβ3 is transformed from low affinity to high affinity state for attachment with its extra cellular ligands. This facilitates binding of αIIbβ3 to fibrinogen, fibrin, von Willebrand Factor (vWF)and fibronectin vitronectin and thrombospondin and promotes platelet aggregation.^9^

αVβ3; Platelet expresses 50-100 αVβ3 receptors on their surface. In the presence of magnesium or manganese, αVβ3 receptors mediate platelet adhesion to vitronectin, fibrinogen, vWF, prothrombin and thrombospondin. Activated αVβ3 also interacts with osteopontin found in atherosclerotic plaques.^10^

***2. Leucine-rich repeats*** ***receptors: ***Leucine-rich repeat (LRR) is a protein structural motif composed of repeating 20-30 amino acids which are rich in the hydrophobic amino acid leucine. LRRmotifs are involved in the formation of protein-protein interactions. LRRmotifs are the most important receptors of platelets that include GPIb-IX-V complex, toll-like receptors (TLR) and matrix metalloproteinases (MMP).

GPIb-IX-Vcomplexis the second most common platelet receptor after integrin αIIbβ3, with approximately 50,000 copies per platelet. GPIb-IX-V complex is instrumental in the initiation and propagation of both hemostasis and thrombosis.^11^

Toll-like receptors (TLR) are present on the surface of platelets in smaller amounts than GPIb-IX-V complex. TLRs are transmemebrane proteins consist of a LRP extracellular domain, a transmembrane region and a Toll-IL-1R domain. Human TLRs are similar to Toll receptor in Drosophila. They receive their name from their similarity to the protein coded by the Toll gene identified in Drosophila in 1985. The gene when mutated made the Drosophila flies look unusual. The researchers were so surprised that they spontaneously shouted out in German "Das istja toll!" that means "That's great!"

Four types of TLRs i.e. 1, 2, 4 and 6 have been identified on platelets. TLRs play an important role in innate immunity by their ability to identify the products of bacteria, viruses, protozoa and fungi. After identification of these products, TLRs activate intracellular signaling pathways to induce an inflammatory response.^12^ TLR-4 promotes platelet-neutrophil interaction and also causes activation of the neutrophils.^13^

Matrix metalloproteinases (MMPs) play a pivotal role in platelet adhesion and aggregation. These include MMP-1, 2, 3, 9 and 14. MMP-1 augments collagen-induced platelet activation mediated by GPVI and α2β1. In resting platelets, MMP-2 exists in inactive form; after platelet activation it becomes translocated to the platelet surface through binding with IIb3 and cleaves CD40 ligand. It also enhances platelet aggregation.^14^

***3. Selectin*** ***s:*** Selectins are a group of receptors having a pivotal role in adhesion. These receptors are not only present on platelets, they are also found on endothelial cells and lymphocytes. Members of selectin family of platelets include P-selectin, C-type lectin-like receptor-2 (CLEC-2) and CD 72.Of the three P-selectin and CLEC-2 are important and well understood.

P-Selectin, also known as CD62P, granule membrane protein (GMP) 140 or platelet activation-dependent granule-external membrane(PADGEM) is a glycoprotein of 140 kDa. It is present in α-granules of resting platelets and is translocated to the plasma membrane after activation.^15^ Platelet activation results in the surface expression of approximately 13,000 P-selectin molecules. P-selectin expression on circulating platelets indicates in vivo activation of platelets. P-selectin attaches neutrophils and monocytes to the platelets and endothelial cells. It also recruits monocyte-derived pro-coagulant microparticles that contain P-selectin glycoprotein ligand-1(PSGL-1) and tissue factor and helps in thrombus formation.^16^

C-type lectin-like receptor-2 (CLEC-2) is a transmembrane platelet receptor for podoplanin and rhodocytin which have the ability to cause platelet aggregation.^17^Podoplaninis present on tumor cells, lymphatic and endothelial cells. Rhodocytin is a protein in the snake venom that has the ability to aggregate platelets.

***4. Tetraspanins*** ***:*** **Tetraspanins are also called tetraspans or the transmembrane 4 superfamily. **This group of membrane proteins contains four membrane-spanning domains. They are considered as the most important signal transducers across the cell membrane. This group includes CD63, CD9 and CD53. CD63 is the most important member of this family.

CD63 or lysosomal membrane-associated glycoprotein-3 (LAMP-3) is a 53 kDa lysosomal membrane protein that appears on the surface of the activated platelets after release reaction.CD63 modulates platelet spreading and platelet tyrosine phosphorylation on immobilized fibrinogen.^18^ CD63 is an extremely reliable marker for in vivo platelet activation.^15^

***5. Transmembrane receptors*** ***: ***This is a major agonist receptor family which is well expressed on the surface of the platelets. These are transmembrane receptors playing an important role in the platelet activation and aggregation. These receptors include ADP and thrombin receptors.

Adenosine Diphosphate (ADP) receptors; ADP is a weak agonist which is secreted from the dense granules of platelets and damaged red cells after stimulation. There are 2 types of purinergic receptors in platelet membrane. One type is guanosine triphosphate coupled protein receptors known as P2Y. The other type of receptor is an ion channel receptor called P2X_1_. These receptors play a pivotal role in platelet activation and aggregation. P2X_1_ binds adenosine triphosphate and mediates extracellular calcium influx leading to alterations in platelet shape.

 ADP receptors have two components i.e. P2Y_1_ and P2Y_12_. ADP is not only an important physiologic agonist; it also augments platelet aggregation induced by other agonists. ADP performs a variety of functions in platelet activation i.e. Ca^2+^ mobilization shape changes, release reaction, production of thromboxane A_2_ (TXA_2_), αIIbβ3 activation and platelet aggregation by attachment with P2Y_1_ receptor.^19^

***6. Prostaglandin receptors*** ***: ***Prostaglandins are lipid derived products that regulate a large number of physiological functions of central nervous system, cardiovascular, gastrointestinal, genitourinary, endocrine, respiratory and immune systems. Platelets have receptors for prostaglandins that play important modulatory functions, thromboxane receptors, prostacyclin(PGI2), PGD2 and PGE2 receptors and PGE2 are important prostaglandin receptors.

Thromboxane receptors; Platelets have thromboxane A_2_/prostaglandin H_2_ (TxA_2_/PGH_2_) receptors with a molecular weight of 57 kDa. These receptors activate phospholipase A_2_ and phospholipase C through signal transduction by G proteins. Following the stimulation by agonists, TXA_2_/PGH_2_ receptors amplify platelet activation by autocrine mechanism.

Prostacyclin receptors (PGI2) are the major inhibitory prostaglandin receptors on platelets. These receptors interact with G proteins to activate adenylatecyclase and bind prostacyclin to keep the platelets in the resting state.^20^

PGE_2_ receptorsinitiate platelet activation at low concentrations of ADP and collagen while higher concentrations of these agonists inhibit PGE_2_ mediated platelet activation. Platelet aggregation is inhibited by the attachment of EP3 receptor to PGE_2_ because of reduced production of cyclic AMP.^21^

***7. Lipid receptors*** ***:*** Platelet activating factor (PAF) receptors are composed of phospholipids. Number of PAF receptors on the platelet surface is approximately 300. These receptors mediate inflammation, anaphylaxis, platelet aggregation and degranulation.^22^

Lysophosphatidic acid receptors (LPL-R) are members of a family of seven transmembrane receptors with a molecular weight of 38-40 kDa. They are expressed on activated platelets and act as autocrine agonists. They bring about shape changes, initiate release reactions and induce platelet aggregation.^23^

***8. Immunoglobulin superfamily receptors*** ***: ***Immunoglobulin superfamily (IgSF) is a large group of cell surface proteins involved in the recognition, binding and adhesion of cells. Molecular structure of these proteins is similar to that of immunoglobulins having a domain known as an immunoglobulin domain or fold. Members of the IgSF include cell surface receptors, co-receptors and co-stimulatory molecules. Immunoglobulin superfamily of platelets receptors comprised of seven members.

GPVI, a major receptor for collagen, is a transmembrane glycoprotein with a molecular weight of 62 kDa.^4^FcRγ is a dimer that forms a high-affinity complex with two molecules of GPVI on the surface of the platelet.^24^

FcγRIIA (CD32) is another member of the immunoglobulin super family with a molecular weight of 40 kDa.^4^It exists in close proximity to the GPIb-IX-V complex. Cross linking of FcγRIIA initiates tyrosine phosphorylation, phosphoinositol metabolism, phospholipase C activation, calcium signaling and cytoskeletal rearrangement.^5^It also mediates signal transduction leading to vWF binding with GPIb. This receptor also mediates integrin αIIbβ3 outside-in signaling.^25^

FcεRI receptors (CD23) cause release of serotonin and regulated upon activation normal T cell expressed and presumably secreted (RANTES) from platelets after activation which plays a role in inflammation. Platelet and T-cell antigen are involved in the differentiation of human cytotoxic T cells; they also mediate platelet adhesion to the damaged endothelium.^26^

Junctional adhesion molecules (JAM): are a family of glycoproteins characterized by two immunoglobulin folds in the extracellular domain. JAM proteins are present in the intercellular junctions of endothelial cells and epithelial cells and also on the surface of leukocytes and platelets. JAM proteins regulate leukocyte, platelet, endothelial cell interactions and tight junction formation in epithelial and endothelial cells. JAM proteins play an important role in platelet activation. JAM are of three types i.e. JAM-1, JAM-2 and JAM-3. Molecular weight of JAM-1 is 32 kDa while that of JAM-2 is 35 kDa. They activate platelets by cross-linking with FcγRIIA.^27^

JAM-3 has a molecular weight of 43 kDa. Each platelet has approximately 1600 copies of JAM-3. It is a transmembrane receptor for the leukocyte integrin Mac -1 that mediates leukocyte–platelet interactions. It has also a role in atherothrombosis.^28^

Platelet-endothelial cell adhesion molecule-1 (PECAM-1, CD31) structurally is a glycoprotein with a molecular weight of 130 kDa. PECAM-1 is found on the surface of platelets, granulocytes and monocytes. Platelet PECAM-1 serves as a regulator of platelet reactivity and thrombosis. Approximately 8,000 PECAM-1 are present on the surface of the platelets.^29^

TREM-Like transcript-1(TLT-1) is found in the platelet α-granules. External domain of this receptor is similar to a family of myeloid cell receptors known as triggering receptors on myeloid cells (TREMs). It can be demonstrated on the surface of the platelets after activation. Cross-linking of TLT-1 with FcεRI increases the calcium signaling.^30^

***9. Tyrosine kinase receptors*** ***:*** Tyrosine kinases receptors are a diverse group of trans-membrane proteins that act as receptors for cytokines, growth factors, hormones and other signaling molecules. Of the 90 unique tyrosine kinase genes identified in the human genome, 58 encode receptor tyrosine kinase proteins. Platelet tyrosine kinase receptors include the following members.

Thrombopoietin receptors(c-mpl, CD110) belong to tyrosine kinase receptor family with a molecular weight of 80-84 kDa.They regulate platelet response to other agonists. Although the number of thrombopoietin receptors is low i.e. 25-224 per platelet, their affinity for thrombopoietin is very high.^31^

Leptin receptors are found on the surface of the platelets; their molecular weight is 130 kDa. They regulate energy storage by fat. Leptin is a 16 kDa protein that regulates energy metabolism.^32^

Tyrosine kinase with immunoglobulin and epidermal growth factor homology-1 receptors (Tie-1) is a protein of 110 kDa molecular weight which are present on the surface of the platelets. Activation of the platelets enhances their expression.^33^

Insulin receptors are also found on platelets. Platelets insulin receptors are functional and metabolic changes occur in them in response to insulin. Binding of insulin to platelets increases the number and activity of adenylatecyclase-linked prostacyclin receptors on their surface.

Platelet-derived growth factor receptors (PDGF) areα-receptors. Binding of PDGF to its receptor initiates tyrosine phosphorylation based signaling cascade.

***10. Miscellaneous platelet membrane receptors*** ***:*** Serotonin receptors; 5-hydroxytryptamin 2A (5-HT2A) is a major receptor. Interaction of 5-HT2A with serotonin initiates calcium signaling. Platelet activation releases serotonin from dense granules that amplify release reaction and platelet aggregation. Serotonin itself cannot cause platelet aggregation but it enhances aggregation induced by other agonists e.g. ADP and thrombin.^34^ Serotonin also causes vasoconstriction of the blood vessels with damaged endothelium and promotes thrombus formation. Serotonin has the ability to attach to a large number of substrates including fibrinogen, vWF, thrombospondin, fibronectin and α_2_-antiplasmin.^35^

CD36 (GPIIIb) is an adhesive glycoprotein with a molecular weight of 88 kDa. There are 20,000 copies of GPIIIb on the surface of each platelet.CD36 facilitates binding of microparticles to the platelets that predisposes to platelet-mediated thrombosis. CD36 also plays an important role in the transportation of long-chain fatty acids and contributes to atherosclerosis, angiogenesis, inflammation and insulin sensitivity leading to diabetes mellitus.^36^

C1q receptors (C1qR) on the platelets are glycoproteins with a molecular weight of 46 kDa. C1qR modulate platelet interactions with collagen and immune complexes at the site of vascular injury, inflammation and atherosclerotic plaques.^37^Surface expression of C1qR is low in resting as well as activated platelets though their number increases after platelet adhesion to immobilized fibrinogen and fibronectin.

Lysosomal-associated membrane proteins 1 and 2 (LAMP-1, CD107a; LAMP-2, CD107b)LAMP-1 and 2 integral membrane glycoproteins are found in the lysosomes and dense granules of platelets and serve as markers of platelet activation. Molecular weight of LAMP-1 is 110 kDa while that of LAMP-2 is 120 kDa.

CD40 ligand; is a transmembrane glycoprotein with a molecular weight of 33 kDa. It belongs to tumor-necrosis factor family (TNF) that is present in the granules in the resting platelets and is rapidly translocated to the platelet surface upon activation. After platelet activation, an 18 kDa soluble fragment of CD40 (CD40L) is released from the platelet surface and circulates in blood in trimeric configuration. This release is mediated by MMP-2.^14^ Since soluble CD40L in plasma originates from activated platelets, it can serve as a marker for in vivo platelet activation. Both platelet-associated CD40 and soluble CD40L stimulate leukocytes to release pro-inflammatory cytokines. It is believed that CD40L may also inhibit endothelial cell migration after vascular injury.^38^

## Authors Contribution

*Muhammad Saboor, Qamar Ayub, and Samina Ilyas *carried out the literature search and wrote the manuscript. *Dr. Moinuddin* reviewed and finalized the article.
